# A Versatile Bonding Method for PDMS and SU-8 and Its Application towards a Multifunctional Microfluidic Device

**DOI:** 10.3390/mi7120230

**Published:** 2016-12-14

**Authors:** Zhen Zhu, Pan Chen, Kegang Liu, Carlos Escobedo

**Affiliations:** 1 Key Laboratory of MEMS of Ministry of Education, Southeast University, Sipailou 2, Nanjing 210096, China; 220141224@seu.edu.cn; 2 Nanomedicine Research Lab CLINAM, University Hospital Basel, Bernoullistrassse 20, Basel CH-4056, Switzerland; kegang.liu@unibas.ch; 3 Department of Chemical Engineering, Queen’s University, 9 Division St., Kingston, ON K7L 3N6, Canada; ce32@queensu.ca

**Keywords:** bonding, PDMS, SU-8, microfluidics, multifunctional integration, cell trapping, negative dielectrophoretic (nDEP), impedance measurement

## Abstract

This paper reports a versatile and irreversible bonding method for poly(dimethylsiloxane) (PDMS) and SU-8. The method is based on epoxide opening and dehydration reactions between surface-modified PDMS and SU-8. A PDMS replica is first activated via the low-cost lab equipment, i.e., the oxygen plasma cleaner or the corona treater. Then both SU-8 and plasma-treated PDMS samples are functionalized using hydrolyzed (3-aminopropyl)triethoxysilane (APTES). Ultimately, the samples are simply brought into contact and heated to enable covalent bonding. The molecular coupling and chemical reactions behind the bonding occurring at the surfaces were characterized by water contact angle measurement and X-ray photoelectron spectroscopy (XPS) analysis. The reliability of bonded PDMS-SU-8 samples was examined by using tensile strength and leakage tests, which revealed a bonding strength of over 1.4 MPa. The presented bonding method was also applied to create a metal-SU-8-PDMS hybrid device, which integrated SU-8 microfluidic structures and microelectrodes. This hybrid system was used for the effective trapping of microparticles on-chip, and the selective releasing and identification of predefined trapped microparticles. The hybrid fabrication approach presented here, based on the PDMS-SU-8 bonding, enables multifunctional integration in complex microfluidic devices.

## 1. Introduction

In microfluidics, poly(dimethylsiloxane) (PDMS) is the most commonly used material to fabricate micro channels [1,2]. Thanks to a soft lithography technique, open PDMS channels can be simply replicated from master molds, and be irreversibly bonded to glass via oxygen plasma activation of sample surfaces [3,4,5,6]. Although PDMS elastomer offers advantages in the fabrication of microfluidic devices, such as ease of use and rapid prototyping, the elasticity of PDMS may cause geometric deformation during the replication, bonding, and fluidic operation at high pressures [7,8,9,10,11]. Any dimensional change in PDMS structures is critical when the specific geometry of devices is seriously concerned. Moreover, it is difficult to precisely align fine PDMS channels with pre-patterned microstructures, such as metal electrodes on the substrate, since alignment equipment, such as mask aligners, cannot be utilized to manipulate elastic PDMS parts [12,13,14]. 

In most cases, SU-8 photoresist is utilized to fabricate masters for PDMS replication [2,4,15]. However, SU-8 is also good for constructing microfluidic structures and has unique properties. First, crosslinked SU-8 has a high Young’s modulus which prevents microchannels from deformation under high hydraulic pressure [16,17]. Second, SU-8 enables wafer-level processes and allows for the precise alignment with pre-patterned functional structures on wafer [18,19]. For these reasons, SU-8 renders microfluidic devices with the potential to integrate multiple functions, such as microelectrodes and actuators, which can be applied in important biological applications, including single-cell studies and biosensing [20,21,22,23]. 

In comparison with PDMS, SU-8 is less prevalent in microfluidics. One major reason is that open SU-8 channels are difficult to be irreversibly sealed with other materials, such as PDMS. Crosslinked SU-8 is thermoset and does not allow for molecular diffusion or entanglements with other materials, which averts its physical bonding to PDMS [24,25]. Recently, several chemical bonding methods based on the amino-epoxy reaction occurred at the interface between PDMS and SU-8 have been reported. One way is by introducing amino groups on PDMS surface under N_2_ plasma [25]. The amino groups then react with the epoxy groups of partially cured SU-8 to form covalent bonding. However, N_2_ plasma is not accessible in most labs, and SU-8 photoresist must be carefully baked to preserve sufficient uncrosslinked polymers for related chemical reactions. Hence, the bonding reliability is highly sensitive to the baking temperature and storage time of SU-8. 

Another common way to introduce amino groups on a PDMS or SU-8 surface as an activated intermediate for bonding, is silanization with aminosilanes, such as (3-aminopropyl)triethoxysilane (APTES) and (3-aminopropyl)trimethoxysilane (APTMS). Aminosilane groups can be directly coupled to hydroxyl groups of plasma-activated PDMS via a dehydration reaction and reserve amino terminals for the following amino-epoxy reaction on the surface of SU-8. Since aminosilane is prone to hydrolyze in moisture or water, Zhang et al. used APTMS in anhydrous toluene solution to modify PDMS surface [26,27]. However, the toxicity of toluene that might remain in PDMS made this method not suitable for biological applications [28]. Ren et al. used pure APTES to functionalize PDMS surface [29]. However, the crosslinking of SU-8 photoresist was carefully controlled by baking the exposed photoresist below 80 °C so that sufficient residuals of epoxy groups can react with the amino groups on PDMS. Moreover, after sample surfaces were contacted, the baking procedure took a long duration by slowly raising the temperature up to 150 °C, keeping for one hour and cooling down naturally, and meanwhile a heavy object was placed on the contacted sample. This bonding protocol, thus, is susceptible to the processing parameters, e.g., the heating rate, temperature, and duration for baking as well as the force applied to the contacted sample. 

In addition to the silanization of PDMS surface, SU-8 can also be silanized with aminosilane, either in vapor phase or in aqueous solution. Talaei et al. performed a vapor-phase silanization by evaporating and depositing APTES molecules on SU-8 surface in a closed chamber [20,23,30]. The silanized SU-8 surface bonded to the plasma-activated PDMS by a dehydration reaction. It should be noted that a plasma power of 400 W was required to treat PDMS in this bonding protocol. Vlachopoulou et al. used APTES in aqueous solution to silanize the plasma-treated SU-8 surface [31]. Then, the APTES-functionalized SU-8 was activated again with oxygen plasma in a RIE machine to form hydroxyl terminals on its surface. After contacting SU-8 with the plasma-treated PDMS, the hydroxyl groups on both surfaces formed strong siloxane bonds via condensation. However, the surface activation of samples in the protocols mentioned above requires strong oxygen plasma, which has to be generated by reactive-ion etching (RIE) or exposure to a plasma asher. The equipment is not always available in most microfluidic labs, thereby restricting the versatility of those bonding methods. 

In this paper, we present a reliable, biocompatible and versatile bonding method for PDMS and SU-8 based on the surface activation of PDMS with oxygen plasma and APTES-involved epoxide opening and dehydration reactions. Compared to the aforementioned methods, any critical parameter or equipment required in the bonding process is avoided herein. For example, SU-8 lithography can be simply processed without additional optimization of post-exposure baking and hard baking processes or low-cost laboratory equipment, i.e., a low-power oxygen plasma cleaner or a handheld corona treater can be used for the plasma activation of PDMS surfaces. Both SU-8 and plasma-treated PDMS samples are functionalized in the aqueous APTES solution, and then are brought into contact and heated to facilitate the covalent bonding. We further characterized the molecule coupling and chemical reactions occurring on the sample surfaces by water contact angle measurement and X-ray photoelectron spectroscopy (XPS) analysis. The bonding strength between PDMS and SU-8 is examined by using tensile strength and leakage tests. Based on this bonding method, we fabricate a metal-SU-8-PDMS archetype hybrid microdevice. The device has an SU-8 channel network structured directly on an electrode-patterned substrate, so that the fluidic structures can be precisely aligned with electrodes underneath via a mask aligner. The microdevice features three functions: (i) hydrodynamic trapping of single microparticles at small orifices that are constructed in SU-8; (ii) selective releasing of trapped microparticles by negative dielectrophoretic (nDEP) forces that are generated from electrodes under the orifices; and (iii) sensitive identification of trapped microparticles by using electrical impedance measurements that are implemented through the same electrodes, but working in a different way. We characterize the device and verify these functions by using monodisperse polystyrene microparticles. 

## 2. Materials and Methods

### 2.1. Surface Modification

**Figure 1:**
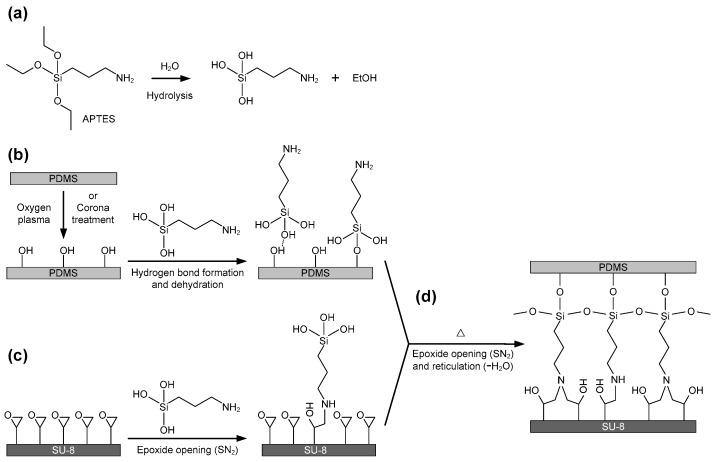
Surface modification and reaction in the poly(dimethylsiloxane) (PDMS)-SU-8 bonding process: (**a**) hydrolysis of APTES reagent in water; (**b**) surface reaction of plasma-activated PDMS in the aqueous (3-aminopropyl)triethoxysilane (APTES) solution. The hydrolysate of APTES molecules is coupled to the silanol groups on the PDMS surface; (**c**) surface reaction of SU-8 in the aqueous APTES solution. The amino groups of APTES molecules react with the epoxy groups on the SU-8 surface (SN_2_ reaction); (**d**) further epoxide opening reaction (SN_2_) and condensation (reticulation, –Si–O–Si– formation) occurred at the sample interfaces when the APTES-functionalized PDMS and SU-8 were contacted and heated.

PDMS (Sylgard^®^ 184 kit), SU-8 3025 photoresist and APTES (99%) were purchased from Dow Corning, MicroChem (Newton, MA, USA) and Sigma Aldrich (‎St. Louis, MO, USA) respectively. Figure 1 schematically shows the possible reactions on the PDMS and SU-8 surfaces for their bonding by using the aqueous APTES solution. First, APTES molecules were hydrolyzed in water to provide respective silanol and ethanol [32,33], as shown in Figure 1a. Second, the PDMS replica was activated to form silanol groups on its surface through an oxygen plasma cleaner (PDC-002-HP, Harrick Plasma, Ithaca, NY, USA) or a laboratory handheld corona treater (BD-20AC, Electro-Technic Products, Chicago, IL, USA). Together with the SU-8 substrate, the plasma-activated PDMS replica was immediately immersed in the aqueous APTES solution. On one hand, the hydrolyzed APTES molecules were attached to the PDMS surface through hydrogen bonds and further underwent a condensation reaction to form the animated PDMS surface, as shown in Figure 1b. On the other hand, the amino groups (–NH_2_) of the hydrolyzed APTES might react with epoxides on the SU-8 surface to obtain a new activated surface with flexible functional chains (epoxide opening, SN_2_ reaction) [34], as shown in Figure 1c. When both the silanized PDMS and SU-8 were ultimately contacted and heated, the amino terminals on the PDMS surface reacted with the residual epoxy groups on the SU-8 surface and the silanol groups on the PDMS surface reacted with each other via the dehydration condensation to form a reticulation of siloxane bonds (–Si–O–Si–) [33]. Therefore, the dehydration condensation and the epoxide opening reaction using APTES as a medium provided a strong bonding between the PDMS and SU-8 surfaces, as shown in Figure 1d.

### 2.2. Bonding Strategy

The bonding strategy of PDMS replica and SU-8 substrate is as follows. (i) To ensure the clean surfaces of PDMS replica and SU-8 substrate, both samples were washed with isopropyl alcohol (IPA) and deionized (DI) water, and then dried with compressed air; (ii) 5% *v*/*v* aqueous APTES solution was made by mixing 3 mL APTES reagent with 57 mL DI water; (iii) The surface activation of PDMS replica was performed in the oxygen plasma cleaner at 30 W for 15 s or under the corona for 30 s; (iv) The plasma-activated PDMS replica and SU-8 substrate were immediately immersed in the 5% *v*/*v* aqueous APTES solution for 20 min; (v) Afterwards, both samples were washed with DI water and dried with filtered compressed air; (vi) The APTES-functionalized PDMS and SU-8 were manually pressed into contact and placed on a hotplate at 90 °C for at least 30 min. Eventually, the PDMS replica and SU-8 substrate were bonded irreversibly.

### 2.3. Surface Analysis

#### 2.3.1. Contact Angle Measurement

We performed the contact angle measurement with a self-made setup. The setup has a *xyz-*positioning stage for placing the sample, and a camera with optical lens facing the side view of the stage. A 10 µL ultrapure water droplet was pipetted onto the sample surface. The *xyz* position of the stage and the focal length of optical lens were adjusted manually to ensure that the camera focuses on the water droplet. Photos of water contact angles were taken and subsequently analyzed with ImageJ software. Eight measurements for each sample surface were performed and averaged.

#### 2.3.2. X-ray Photoelectron Spectrometry (XPS) Analysis

XPS analysis was performed by using an X-ray photoelectron spectroscopy (PHI 5000 VersaProbe, ULVAC-PHI, Chigasaki, Japan) equipped with a monochromatic aluminum Kα X-ray source. During the data acquisition, the peak of interest (C, O, N, and Si) on the spectra was further scanned at high resolution. 

### 2.4. Bonding Strength Analysis

#### 2.4.1. Tensile Strength Test

**Figure 2:**
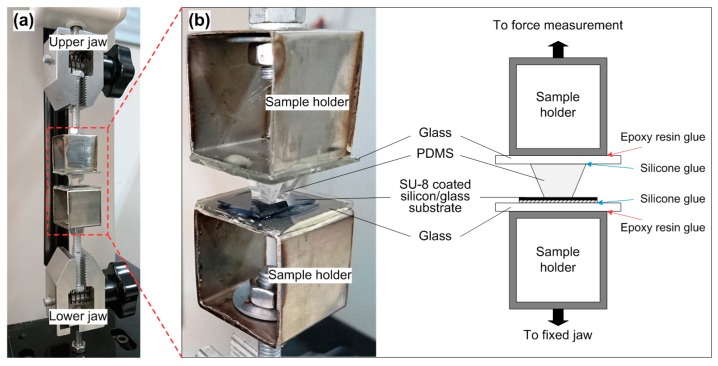
Setup of the tensile strength test. (**a**) Setup of the tensile tester. It has an immobile jaw at the lower position and a movable jaw connected to a force sensor at the upper position. The sample is fixed on the jaws through screws; (**b**) assembled sample in the tester. The sample holders were first glued with two bare glass slides. The bonded PDMS-SU-8 sample was then sandwiched between the two glass slides using silicone glue.

The bonding strength between PDMS and SU-8 was evaluated by using a tensile tester (HSV-500, Handpi, Yueqing, China). The setup of the tensile tester and the assembled sample are shown in Figure 2. The sample was prepared by bonding a PDMS replica at the center of an SU-8-coated silicon substrate. The SU-8 layer has a thickness of 20 µm, and underwent soft baking, UV exposure, post-exposure baking, and hard baking. The contact area between PDMS and SU-8 was 5 × 5 mm^2^, determined by the contact surface of PDMS replica. Two steel sample holders were used to sandwich the bonded sample in between. A glass slice was glued on each sample holder using epoxy resin glue. Then, the upper part of PDMS replica and the backside of silicon substrate were glued on glass slices of the two holders using silicone glue, respectively. The sandwiched sample and its holders were next fixed on the jaws of the tensile tester via screws. During the tensile test, the upper jaw moved upwards at a uniform speed of 1.5 mm/s, and the tensile force was recorded simultaneously until the bonded sample broke. The tensile strength of the tested sample was calculated by dividing the contact area 25 mm^2^ into the measured force when the sample broke.

#### 2.4.2. Leakage Test

A leakage test was performed by infusing a red dye solution into the channel of the bonded chip. The channel was constructed in SU-8 photoresist, and bonded to a flat PDMS sheet with punched holes for fluidic connection. The diameter of those holes on PDMS is 0.75 mm. The SU-8 channel has a width of 200 µm, a height of 30 µm, and a length of 25 mm. Two types of chips were fabricated: single- and double-ended ones. The single-ended chip had only one punched hole, which served as the inlet for fluid injection and was aligned to one end of the channel during bonding. In comparison, the double-ended chip had two punched holes, which were aligned to both ends of the channel and worked as the inlet and outlet for fluid perfusion. Polytetrafluoroethylene (PTFE) tubing was directly inserted in the punched holes of the PDMS layer. The inserted tubing had an inner diameter of 0.5 mm and an outer diameter of 1 mm, so it could be held in place due to the elasticity of PDMS. A syringe, filled with a red dye solution, was connected to the inlet through the tubing and infused the red dye into the channel by using a syringe pump (neMESYS, Cetoni, Korbußen, Germany). The flow rate of the red dye solution was precisely controlled with the syringe pump and was increased gradually until the fluid leakage occurred at the inlet.

### 2.5. Fabrication of the Metal-SU-8-PDMS Hybrid Device

The microfluidic device shown in Section 3.3 was fabricated by using a metal-SU-8-PDMS hybrid process, which was described previously [20,23]. Briefly, a 4-inch glass wafer was used as a substrate, where platinum (Pt) electrodes were directly patterned through a lift-off process. Then, a silicon nitride layer was deposited on the whole wafer to passivate all metal lines, and was etched to define the opening of electrodes and electric contact pads. A 30 µm-thick layer of SU-8 photoresist was then spin-coated directly on top of the silicon nitride layer and lithographed to construct the microfluidic network. As the fluidic structures were patterned in SU-8, we exploited the use of the mask aligner which ensures the precise alignment between SU-8 structures and Pt electrodes on the glass substrate. Consequently, each diced metal-SU-8 chip was permanently sealed with a PDMS sheet using this PDMS-SU-8 bonding method. Prior to bonding, holes were punched on the PDMS sheet for the fluidic connections. 

## 3. Results and Discussion

### 3.1. Characterization of PDMS and SU-8 Surfaces

**Figure 3:**
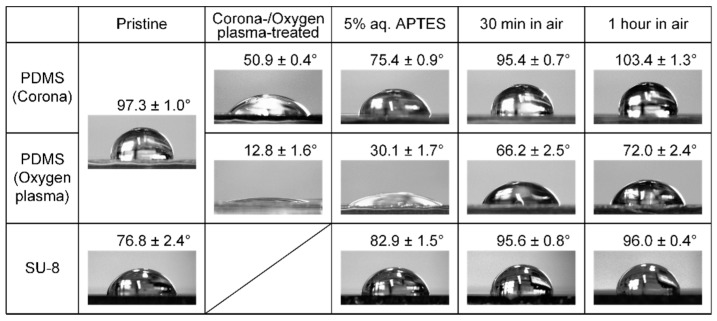
Water contact angles measured on the pristine PDMS and SU-8, corona- and oxygen plasma-treated PDMS, APTES-modified PDMS and SU-8, as well as 30-min- and 1-h-aged PDMS and SU-8 after APTES functionalization.

We first measured the water contact angles to characterize the surfaces of PDMS replica and SU-8 substrate after each step of functionalization. Figure 3 shows the water contact angles of the untreated PDMS and SU-8, corona- and oxygen plasma-treated PDMS, and APTES-modified PDMS and SU-8. After the surface activation, the contact angle immediately dropped from 97.3° to 50.9° for the corona-treated PDMS, and from 97.3° to 12.8° for the oxygen plasma-treated PDMS. The drastic decrease of contact angles indicated that the hydrophobic surface of pristine PDMS became hydrophilic owing to the hydroxyl terminals on the plasma-activated PDMS surface. It is important to note that the corona-treated PDMS has a larger contact angle, implying less density of hydroxyl groups on the PDMS surface, compared to the oxygen plasma-treated PDMS. The result can be attributed to weaker air plasma generated by the corona treater than that from the oxygen plasma cleaner. After immersing the plasma-activated PDMS in the aqueous APTES solution for 20 min, we observed a remarkable increase of the water contact angle of PDMS samples (75.4° and 30.1° for the corona- and the oxygen plasma-treated PDMS, respectively). The result implied the reduction of the surface hydrophilicity due to the surface salinization with APTES. The subsequent dehydration reaction of hydroxyl groups between plasma-activated PDMS and hydrolyzed APTES formed animated intermediates on the surface, and meanwhile the surface hydrophilicity was reduced. For SU-8 surface, the contact angle increased slightly from 76.8° to 82.9° after the APTES treatment. This phenomenon can be explained by the reaction between a small portion of SU-8 epoxy groups and the amino terminals of APTES molecules. Moreover, from the measured contact angle, we observed that the hydrophobicity of sample surfaces recovered after aging the silanized PDMS and SU-8 samples in air for 30 min and 1 h. The surface recovery has significant influence on the bonding strength, which is discussed in the next subsection. 

**Figure 4:**
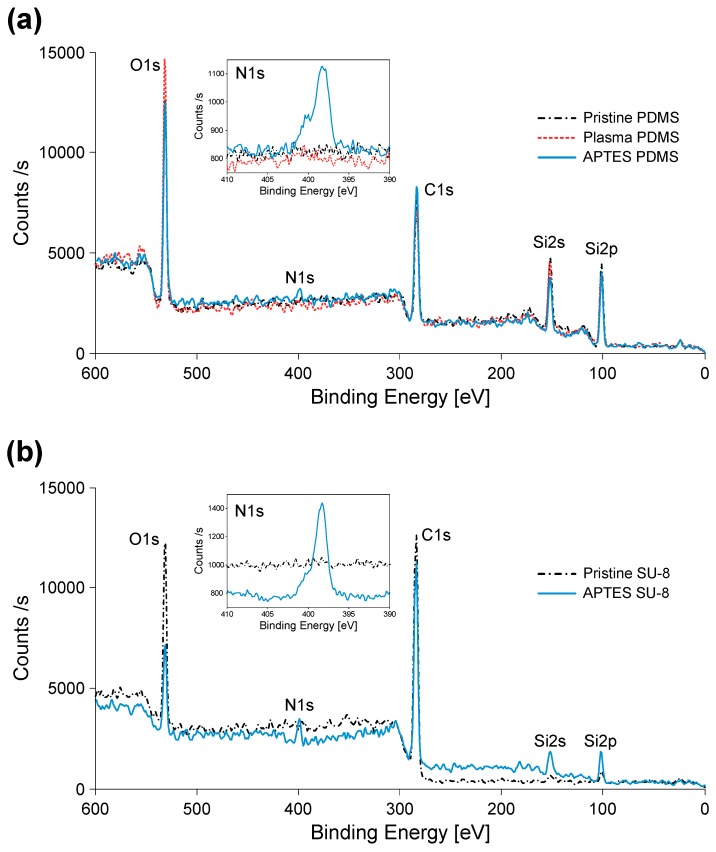
XPS of PDMS and SU-8: (**a**) pristine, plasma-activated, and APTES-modified PDMS; and (**b**) pristine and APTES-modified SU-8.

In order to verify that the APTES molecules were successfully tethered to the sample surfaces, the surface chemicals of PDMS and SU-8 were analyzed by using XPS. Figure 4a shows the XPS spectra of the pristine, plasma-activated, and APTES-modified PDMS. Among all the elemental peaks, the N 1s peak presented for the APTES-modified PDMS, indicating the successful binding of APTES molecules on the PDMS surface. Figure 4b shows the XPS spectra of the pristine and APTES-modified SU-8. The presence of N 1s, Si 2s, and Si 2p peaks for the APTES-modified SU-8 referred to the successful reaction between the amino functional groups of hydrolyzed APTES and the epoxy groups of SU-8, since the pristine SU-8 has no elements of N and Si.

### 3.2. Bonding Strength

**Figure 5:**
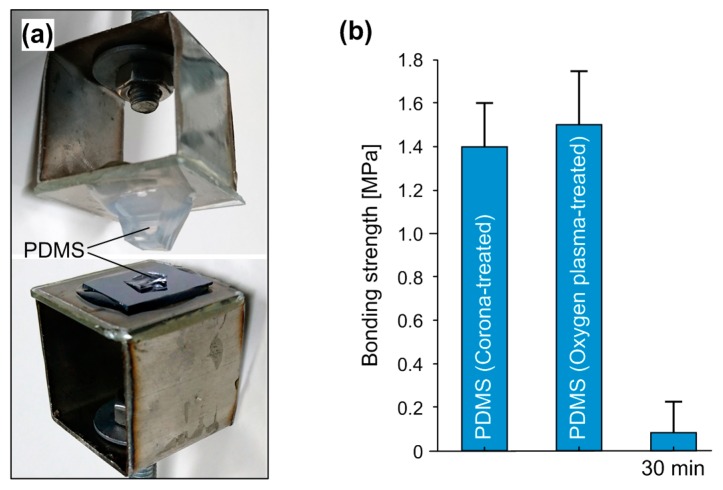
Tensile strength test for the PDMS-SU-8 bonding. (**a**) Photo of a broken sample after the tensile test; (**b**) bonding strength of samples treated under different conditions: corona-activated PDMS, oxygen plasma-activated PDMS and 30-min-aged PDMS and SU-8 after APTES silanization. Error bars represent the standard deviation of seven measured samples under each condition.

The bonding strength between PDMS replica and SU-8 substrate was quantitatively evaluated by using a tensile strength tester (Figure 2a). Each bonded PDMS-SU-8 sample (Figure 2b) was assembled in the following way: Two glass slides were firmly glued on the sample holders with epoxy resin glue first, and the bonded sample was then sandwiched between the glass slides with silicone glue. After each test, the silicone glue was easily scraped off the smooth glass so that the sample holders were reused. In the tensile strength test, we observed that almost all samples were broken in the PDMS replica, rather than on the contact surface of PDMS and SU-8. Figure 5a shows a photo of a broken sample after the tensile strength test. We can clearly see the split PDMS replica and some PDMS residuals on the SU-8 substrate. The result can be explained by the higher bonding strength between PDMS and SU-8 than the breaking point of cured PDMS materials. Figure 5b shows the measured bonding strength of samples treated under different conditions. The bonded PDMS-SU-8 samples with the corona- and oxygen plasma-activated PDMS exhibited high bonding strength over 1.4 MPa. Essentially, the actual bonding strength between PDMS and SU-8 was much higher than the recorded value from the tensile test since the breaking interface of samples always occurred in PDMS. Moreover, we evaluated the influence of aging on the bonding strength by exposing the APTES-functionalized samples in air for 30 min. The low value (0.1 MPa) of bonding strength implied that the 30-min-aged samples were hardly bonded. The result suggests that the immediate contact of functionalized PDMS and SU-8 samples should be carried out during the bonding process. 

**Figure 6:**
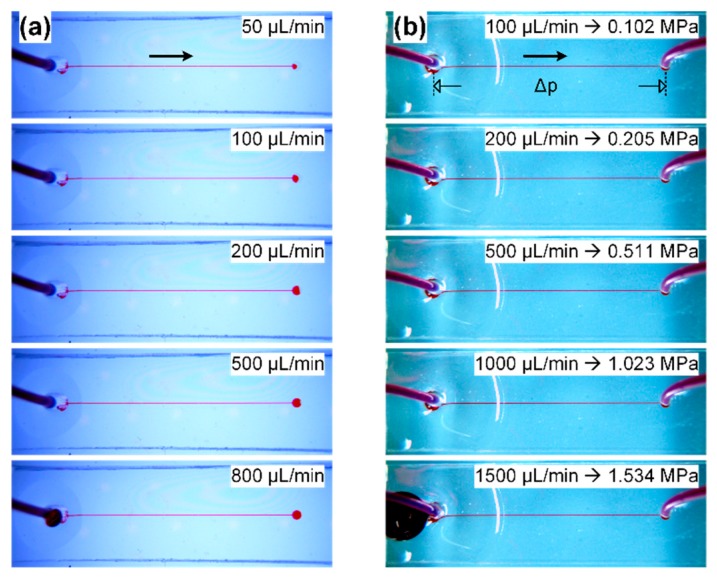
Liquid leakage test for the bonded PDMS-SU-8 channels. (**a**) Single-ended channel infused with a red dye solution. The flow rate was increased from 50 µL/min to 800 µL/min, at which point the red dye leaked out of the inlet; (**b**) double-ended channel infused with a red dye solution. The flow rate was increased up to 1500 µL/min when the red dye leaked out of the inlet. The hydraulic pressure from the channel inlet to the outlet, Δ*p*, was calculated by using Hagen-Poiseuille’s equation, according to the corresponding flow rate and the geometric parameters of the channel.

To investigate the stability of microfluidic channels bonded using this method, we examined the occurrence of delamination and liquid leakage between PDMS and SU-8 under high flow-rate perfusion. Figure 6 shows the photos of microfluidic channels injected with a red dye solution at various flow rates increasing from 50 µL/min to 1500 µL/min. The flow rate stopped increasing until the red dye leaked out of the channel inlet. The liquid leakage was attributed to the direct insertion of PTFE tubing into the inlet holes, which expanded under high hydraulic pressure. We also calculated the hydraulic pressure corresponding to the applied flow rate by using the well-known Hagen-Poiseuille’s equation [35], as shown in Figure 6b. In the test, all the microfluidic channels were kept intact, and no dissemination of red dye solution into the bonded interface was observed along the channels. The result demonstrates that there is no delamination or liquid leakage on the bonded PDMS and SU-8 channels even when the infused liquid has a high flow rate of 1500 µL/min, and the bonded PDMS-SU-8 channels can endure the hydraulic pressure of around 1.5 MPa. 

### 3.3. Towards Multifunctional Application of the Metal-SU-8-PDMS Hybrid Device

**Figure 7:**
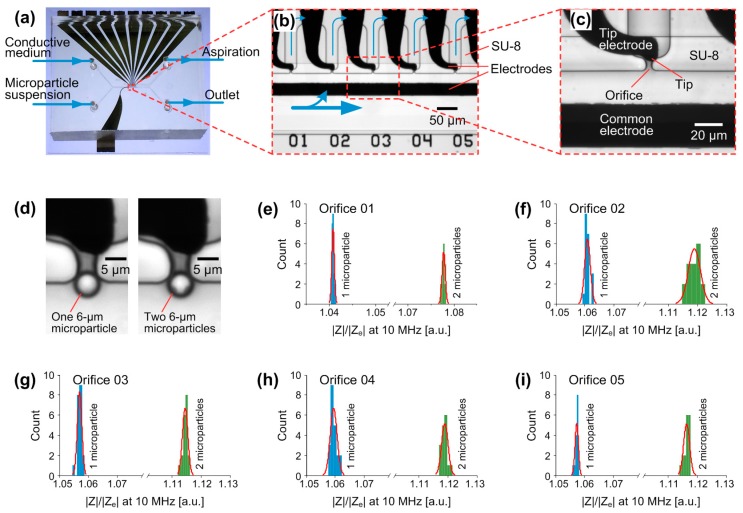
A metal-SU-8-PDMS hybrid device for multifunctional application. (**a**) Photo of the device indicated with the fluidic connections; (**b**) close-up of the device showing five orifices (01–05) and respective electrodes. The fluidic structures, patterned in SU-8, were precisely aligned with Pt electrodes. Blue arrows indicate the flow directions during the microparticle trapping; (**c**) close-up of a unit for microparticle trapping, nDEP releasing and local impedance measurement. The tip of the tip electrode is right situated under the orifice; (**d**) micrographs of one trapped microparticle and two vertically stacked microparticles. The diameter of microparticles used in this work is 6 µm; (**e**–**i**) identification of single and two vertically stacked microparticle(s) at the orifices 01 to 05 through impedance measurements. |Z| and $\left|Z_{e}\right|$ are the impedance magnitudes from measuring the trapped microparticle(s) and the empty orifice, respectively.

Taking advantage of the PDMS-SU-8 bonding, complex microfluidic devices towards multifunctional application can be realized. Here, we designed a microfluidic device that integrated multiple functions of trapping, releasing and identification of single microparticles. The device was fabricated through a metal-SU-8-PDMS hybrid process (see Section 2.5 for details). Figure 7a shows the photo of the fabricated device. On the glass substrate, platinum and a silicon nitride passivation layer was patterned to define the opening of electrodes and contact pads. Those electrodes feature two functions: One as addressable electrodes for the selective releasing of trapped microparticles by using nDEP forces [20], the other as the localized electrodes for the identification of trapped microparticles by using electrical impedance measurement [23]. In order to ensure the successful releasing and the sensitive identification of microparticles, the electrodes have to be accurately positioned at the microparticle-trapping orifices. Therefore, the microfluidic structures were directly patterned in SU-8 photoresist and was precisely aligned with electrodes via a mask aligner. In the end, the microfluidic network on each diced metal-SU-8 chip was sealed with a PDMS sheet by using the PDMS-SU-8 bonding method.

#### 3.3.1. Trapping of Microparticles by Hydrodynamic Forces

The function of microparticle trapping by hydrodynamic forces has been presented in our previous studies [20,23]. The microfluidic network in the device consists of two channels and ten orifices in between (Figure 7a–c). A main channel, located in the center of the device, has two punched holes as inlets for the infusion of medium and microparticle suspension. A side channel, located beside the main channel, connects to an outlet hole where the aspiration is applied via a pressure controller. Orifices with a width of about 4 µm are positioned between the two channels. Once sufficient aspiration is applied to the side channel, microparticles in the main channel can be driven to flow towards the orifices. As the employed microparticles have a diameter of 6 µm, which is greater than the orifice width, they can be captured at the orifices (Figure 7d).

#### 3.3.2. Addressable Releasing of Trapped Microparticles by Negative Dielectrophoretic (nDEP) Forces 

When particles, such as microparticles or cells, are subjected to a non-uniform electric field, they will be polarized and dielectrophoretic (DEP) forces will be generated and exerted on the particles [36,37]. DEP forces can either attract particles towards high field regions (positive DEP, pDEP) or repel particles from high field regions (nDEP), depending on the frequency of applied alternating current (AC) voltage and the relative polarizability of particles and surrounding medium [38]. Thus, DEP forces can be used to manipulate particles in suspension [39]. 

In the microfluidic device, each tip electrode targets one microparticle-trapping orifice with its tip located right under the orifice, and a common electrode located in the main channel is grounded (Figure 7b,c). Once one of the ten tip electrodes is applied with an AC signal, a non-uniform electric field is created between the electrode tip and the grounded common electrode. By choosing the proper conductivity of the medium and frequency of the applied signal, nDEP forces can be generated to release the trapped microparticles at the orifice. The strength of nDEP forces is highly affected by the gradient of electric field, which has a maximum gradient around the electrode tip. Therefore, the microparticle-trapping orifice is designed to be close to the tip of each electrode. It is the reason that the microfluidic structures have to be precisely aligned with the pre-patterned electrodes during fabrication. Moreover, microparticles captured at different orifices can be selectively released by activating the respective tip electrode, thereby achieving the addressable releasing of trapped microparticles. For details about this result, we refer you to our previously published work [20].

#### 3.3.3. Identifying the Trapped Microparticles through Microfluidic Electrical Impedance Spectroscopy (EIS)

Since the fluidic channel is much higher than the microparticle diameter, multiple microparticles can be vertically captured above each other at the orifice (Figure 7d). From the bright-field images with an inverted microscopy (IX 81, Olympus, Tokyo, Japan), it is difficult to distinguish the single microparticle from the vertically stacked multiple microparticles. In our previous study, we integrated the electrical impedance spectroscopy (EIS) to measure trapped microparticles via parallel electrodes and to identify the size and number of microparticles [23,40]. The parallel configuration of electrodes near the microparticle-trapping orifices was designed to improve the sensitivity of impedance measurement, but eliminated the function of microparticle releasing by using nDEP forces. 

In the microfluidic device here, EIS was implemented by sharing the tip electrodes and the common electrode with the use of DEP generation. In comparison, the common electrode served as a stimulus electrode and each tip electrode was used to record the resulting signal for EIS. During the impedance measurement, an AC voltage (1 V_p_, 10 MHz) from an impedance spectroscope (HF2IS, Zurich Instruments, Zürich, Switzerland) was applied to the common electrode. The resulting current was then received by the respective tip electrode and converted to a voltage signal via a current amplifier (HF2CA, Zurich Instruments), and finally was recorded by the impedance spectroscope. 

We performed the impedance measurements of trapped single and two 6 µm polystyrene microparticle(s) and the empty orifice each time after releasing the trapped microparticle(s). Signals from measuring the empty orifice were used to normalize the recorded impedance magnitudes of trapped microparticle(s). Figure 7e–i show the statistic results of measuring microparticle(s) trapped at the orifices 01 to 05. Based on the recorded impedance signals, we can clearly identify the number of microparticle(s), i.e., one or two, trapped at each orifice. The result demonstrates that the tip electrodes are sensitive enough to detect the number of vertically stacked microparticle(s). 

## 4. Conclusions

We have presented a facile, versatile and reliable bonding method for poly(dimethylsiloxane) (PDMS) and SU-8 based on the surface modification with (3-aminopropyl)triethoxysilane (APTES) hydrolysate. During the bonding process, both plasma-activated PDMS and pristine SU-8 substrate were immersed in the aqueous APTES solution for silanization, and then the functionalized samples were immediately contacted and heated to form the siloxane reticulation, thereby facilitating the covalent bonding. The surface characterization of PDMS and SU-8 samples through water contact angle measurement and XPS analysis has validated the successful coupling of APTES molecules on sample surfaces. The tensile test has shown very strong bonding strength between PDMS and SU-8. Even when using a low-cost corona treater for plasma activation of PDMS, the bonding strength reached 1.4 MPa. In comparison, the conventionally PDMS-glass or PDMS-PDMS bonding methods based on the plasma treatment of sample surfaces have exhibited a much lower bonding strength of about 0.5 MPa (see Supplementary Table S1). The leakage test has proved that microfluidic channels bonded by using this method can withstand high hydraulic pressure (>1.5 MPa) from the infused liquid (>1500 µL/min). 

Furthermore, we have summarized the PDMS-SU-8 bonding methods reported previously and listed the critical details of their bonding processes and the tested bonding strength in Supplementary Table S2. Compared to the previously published methods, this PDMS-SU-8 bonding method has shown several advantages: (i) Simple and low-cost equipment in common labs, such as the widely used Harrick oxygen plasma cleaner and the handheld corona treater, was used to perform the plasma activation of PDMS surfaces. No cleanroom equipment, such as reactive-ion etching (RIE) and plasma asher, or N_2_ plasma system was required; (ii) No toxic reagent, such as toluene, was employed in the silanization process of samples, thereby ensuring the biocompatibility of bonded devices; (iii) SU-8 photolithography was processed as the manufacture’s datasheet recommends, even with hard baking to further crosslink the photoresist, thus leading to the versatility of this bonding method for any SU-8-patterned substrate. Additional optimization of the post-exposure baking temperature and time during SU-8 process was averted; (iv) The contacted sample was simply heated for 30 min on a hotplate without increasing or decreasing the temperature gradually or applying a force to the top, and thus the procedure of post-contact baking was simplified and the time consumption was reduced; (v) The bonded microfluidic channels endured a high hydraulic pressure above 1.5 MPa. The tensile test also exhibited strong bonding strength, which was higher than the fracture strength of bulk PDMS material (about 1.5 MPa). Therefore, we have demonstrated that this PDMS-SU-8 bonding method features the reliability, biocompatibility and versatility necessary to fabricate complex microfluidic devices with fine SU-8 microstructures and irreversible PDMS sealing.

Benefiting from this bonding method, we have proposed a hybrid metal-SU-8-PDMS microfluidic device, which has integrated three functions: (i) trapping microparticles; (ii) selectively releasing microparticles by negative dielectrophoretic (nDEP) forces; and (iii) identifying the trapped microparticles by impedance measurements. SU-8 fluidic structures have been precisely aligned with metal electrodes that have been pre-patterned on the substrate. Microparticles, employed in the characterization of the device, can be trapped at each SU-8 orifice and released by nDEP forces that are generated from the AC voltage applied between the respective tip electrode and common electrode. Local impedance measurement of trapped microparticles has been performed via the very same electrodes, but in a different regime. Single and two vertically stacked microparticles can be identified successfully according to the measured impedance signals. Therefore, sophisticated manipulation and sensitive impedance detection of microparticles have been realized. Such a hybrid fabrication process based on the PDMS-SU-8 bonding will facilitate the integration of multiple functions into complex microfluidic devices, which potentially offer suitable platforms for the manipulation and electrical impedance detection of single cells. 

## Supplementary Materials

The following are available online at www.mdpi.com/2072-666X/7/12/230/s1, Table S1: bonding strengths between PDMS and glass/PDMS using plasma activation reported in different literatures, Table S2: various bonding methods between PDMS and SU-8 and evaluation of bonding strength by using different methods title.
