# Supplementary material for: A Versatile Bonding Method for PDMS and SU-8 and Its Application towards a Multifunctional Microfluidic Device
Source: Micromachines (Basel). 2016 Dec 14;7(12):230. doi: 10.3390/mi7120230 (PMC6190230; doi:10.3390/mi7120230)
Supplement: Supplementary file 1 [file micromachines-07-00230-s001.pdf]

Supplementary Materials: A Versatile Bonding Method for PDMS and SU-8 and Its Application towards a Multifunctional Microfluidic Device

Zhen Zhu, Pan Chen, Kegang Liu and Carlos Escobedo

Table S1. Bonding strengths between PDMS and glass/PDMS using plasma activation reported in different literatures.

Reference	Surface Treatment of PDMS-Glass/PDMS-PDMS Bonding	Bonding Strength Evaluated by Different Methods		
		Burst Test by Air (kPa)	Shear Strength Test (kPa)	Tensile Strength Test (kPa)
[1]<br>[2]<br>[3]	PDMS-glass/PDMS-PDMS: O_2 plasma (18 W, 60 s)	NA	NA	207–335
[4]	PDMS-glass: O_2 plasma (200 W, 300 s)	NA	maximum 74	NA
[5]	PDMS-glass/PDMS-PDMS: O_2 plasma (20 W, 30 s)	maximum 510/400	NA	NA
[6]	PDMS-PDMS: Corona (30 s) or O_2 plasma (20 W, 30 s)	average 290/300	NA	NA
[7]	PDMS-PDMS: O_2 plasma (100 W, 60 s)	NA	NA	406

Table S2. Various bonding methods between PDMS and SU-8 and evaluation of bonding strength by using different methods.

Reference	PDMS-SU-8 Bonding Method			Bonding Strength Evaluated by Different Methods		
	Treatment of PDMS	Treatment of SU-8	Post-Contact Treatment	Hydraulic Pressure (kPa)	Shear Strength (kPa)	Tensile Strength (kPa)
This work	1. O_2 plasma (30 W, 15 s) or corona (30 s);<br>2. Aqueous APTES solution (5% *v*/*v*, 20 min)	1. With or without hard baking;<br>2. Aqueous APTES solution (5% *v*/*v*, 20 min)	1. Heating at 90 °C for 30 min	1534	NA	>fraction strength of bulk PDMS
[8]<br>[9]<br>[10]	1. O_2 plasma (400 W, 15 s)	1. With or without hard baking<br>2. Vapor-phase APTES deposition in a closed chamber for 2 h	1. Applying ~0.5 kg/cm² weight to the top;<br>2. Heating at 70 °C for 10 min	NA	NA	NA
[11]	1. N_2 plasma (30 W, 120–240 s)	1. Without hard baking	1. Heating at 100 °C for 30 min	average 990	>fraction strength of bulk PDMS	NA
[12]	1. O_2 plasma (30 W);<br>2. Toluene APTMS solution (0.1–1.0 wt %)	1. Without hard baking	1. Heating at 80–100 °C for 30 min	average 910	NA	NA
[13]	1. O_2 plasma (50 W, 30 s);<br>2. Pure APTES (99%, 5 min)	1. Post-exposure baking temperature lower than 80 °C;<br>2. Without hard baking	1. Applying 2 N/cm² to the top;<br>2. Heating in an oven with a ramp of 2 °C/min to 150 °C (~1 h);<br>3. Keeping at 150 °C for 1 h;<br>4. Cooling down to 30 °C natually (~1 h)	NA	NA	~fraction strength of bulk PDMS

References

1. Chaudhury, M.K.; Whitesides, G.M. Direct measurement of interfacial interactions between semispherical lenses and flat sheets of poly(dimethylsiloxane) and their chemical derivatives. *Langmuir* **1991**, *7*, 1013–1025.
2. Duffy, D.C.; McDonald, J.C.; Schueller, O.J.A.; Whitesides, G.M. Rapid prototyping of microfluidic systems in poly (dimethylsiloxane). *Anal. Chem.* **1998**, *70*, 4974–4984.
3. McDonald, J.C.; Duffy, D.C.; Anderson, J.R.; Chiu, D.T.; Wu, H.; Schueller, O.J.A.; Whitesides, G.M. Fabrication of microfluidic systems in poly (dimethylsiloxane). *Electrophoresis* **2000**, *21*, 27–40.
4. Xiong, L.; Chen, P.; Zhou, Q. Adhesion promotion between PDMS and glass by oxygen plasma pre-treatment. *J. Adhes. Sci. Technol.* **2014**, *28*, 1046–1054.
5. Bhattacharya, S.; Datta, A.; Berg, J.M.; Gangopadhyay, S. Studies on surface wettability of poly(dimethyl) siloxane (PDMS) and glass under oxygen-plasma treatment and correlation with bond strength. *J. Microelectromech. Syst.* **2005**, *14*, 590–597.
6. Eddings, M.A.; Johnson, M.A.; Gale, B.K. Determining the optimal PDMS–PDMS bonding technique for microfluidic devices. *J. Micromech. Microeng.* **2008**, *18*, 067001.
7. Vlachopoulou, M.E.; Tserepi, A.; Pavli, P.; Argitis, P.; Sanopoulou, M.; Misiakos, K. A low temperature surface modification assisted method for bonding plastic substrates. *J. Micromech. Microeng.* **2009**, *19*, 015007.
8. Zhu, Z.; Frey, O.; Ottoz, D.S.; Rudolf, F.; Hierlemann, A. Microfluidic single-cell cultivation chip with controllable immobilization and selective release of yeast cells. *Lab Chip* **2012**, *12*, 906–915.
9. Zhu, Z.; Frey, O.; Haandbaek, N.; Franke, F.; Rudolf, F.; Hierlemann, A. Time-lapse electrical impedance spectroscopy for monitoring the cell cycle of single immobilized *S. pombe* cells. *Sci. Rep.* **2015**, *5*, 17180.
10. Talaei, S.; Frey, O.; van der Wal, P.D.; de Rooij, N.F.; Koudelka-Hep, M. Hybrid microfluidic cartridge formed by irreversible bonding of SU-8 and PDMS for multi-layer flow applications. *Procedia Chem.* **2009**, *1*, 381–384.
11. Zhang, Z.; Zhao, P.; Xiao, G.; Watts, B.R.; Xu, C. Sealing SU-8 microfluidic channels using PDMS. *Biomicrofluidics* **2011**, *5*, 046503.
12. Zhang, Z.; Zhao, P.; Xiao, G. The fabrication of polymer microfluidic devices using a solid-to-solid interfacial polyaddition. *Polymer* **2009**, *50*, 5358–5361.
13. Ren, Y.; Huang, S.-H.; Mosser, S.; Heuschkel, M.; Bertsch, A.; Fraering, P.; Chen, J.-J.; Renaud, P. A Simple and Reliable PDMS and SU-8 Irreversible Bonding Method and Its Application on a Microfluidic-MEA Device for Neuroscience Research. *Micromachines* **2015**, *6*, 1465.
